# Seropositivity for SARS-CoV-2 in a General Population: How Specific Is the Diagnostic?

**DOI:** 10.2188/jea.JE20220151

**Published:** 2023-01-05

**Authors:** Zhaoqing Lyu, Tomoko Fujitani, Kouji H. Harada

**Affiliations:** Department of Health Environmental Sciences, Kyoto University Graduate School of Medicine, Kyoto, Japan

**Keywords:** seroprevalence, SARS-CoV-2, specificity, IgG, immunological response

We read the article by Sanada et al in the Journal of Epidemiology^1^ with great interest. The authors surveyed the seroprevalence of immunoglobulin G (IgG) against SARS-CoV-2 among hospital visitors from September 2020 to March 2021 and found an estimate of 3.40% seropositivity in the Tokyo area. This was 3.9-fold higher than polymerase chain reaction (PCR)-based cases of novel coronavirus disease 2019 (COVID-19).

We have comments on this study regarding the specificity of testing and target proteins. The authors employed two chemiluminescence immunoassay kits (iFlash–SARS-CoV-2 IgG kit and iFlash–SARS-CoV-2 IgG-S1 kit). They are validated better than previous point-of-care testing.^2^ In the methods section, the authors claimed that the diagnostics showed 100% specificity that was calculated in limited sample numbers (around 100) in the cited literature.^3^ The authors also conducted the test by YHLO S1-IgG in PCR-negative subjects (*n* = 163) and found no positive sample. The point estimate of false positive rate (1-specificity) is 0% but the 95% confidence interval is 0–2.3%. The confidence interval should be considered for a low prevalence situation.

The authors used two test kits and found a number of single positive samples (Figure 2 of the article).^1^ The authors stated that “although the iFlash–SARSCoV-2 IgG kit detects anti-N and anti-S antibodies (YHLO IgG), it primarily detects anti-N antibodies”.^1^ If this is correct, the samples with a single positive result only contained either anti-S1 or anti-N antibody. Is this possible? Immunological responses in COVID-19 patients showed elevated IgG antibodies against the N protein, the S1 subunit of the spike protein, and the receptor-binding domain of the spike protein of SARS-CoV-2.^4^ It is possible that they were false positives. The specificity of anti-N antibody is less than that of anti-S antibody because of cross-reactivity with other coronaviruses.^5^ Validation in pre-COVID-19 samples would help the estimation of non-specific cross-reactions.^6^ In addition, the authors conducted the survey from September 1, 2020, to March 31, 2021, and vaccination for COVID-19, which induces anti-S antibodies, began in Japan in 2021. The vaccination status of the participants was not confirmed. It may contaminate the seroprevalence rate.

This is a minor point, but the method to detect anti-N protein antibody titer was not provided in eFigure 1 of the article.^1^
